# Modifications and functional genomics of human transfer RNA

**DOI:** 10.1038/s41422-018-0013-y

**Published:** 2018-02-20

**Authors:** Tao Pan

**Affiliations:** Department of Biochemistry and Molecular Biology, University of Chicago, Chicago, IL 60637 USA

## Abstract

Transfer RNA (tRNA) is present at tens of millions of transcripts in a human cell and is the most abundant RNA in moles among all cellular RNAs. tRNA is also the most extensively modified RNA with, on an average, 13 modifications per molecule. The primary function of tRNA as the adaptor of amino acids and the genetic code in protein synthesis is well known. tRNA modifications play multi-faceted roles in decoding and other cellular processes. The abundance, modification, and aminoacylation (charging) levels of tRNAs contribute to mRNA decoding in ways that reflect the cell type and its environment; however, how these factors work together to maximize translation efficiency remains to be understood. tRNAs also interact with many proteins not involved in translation and this may coordinate translation activity and other processes in the cell. This review focuses on the modifications and the functional genomics of human tRNA and discusses future perspectives on the explorations of human tRNA biology.

## Introduction

Together with ribosomal RNA (rRNA) and messenger RNA (mRNA), tRNA is among the three types of RNA presented to students of any introductory biology course. The primary function of tRNA, as the adaptor molecule for amino acid identity and the genetic code, is well understood. The other property of tRNA sometimes briefly mentioned in introductory biology is the presence of RNA modification. Beyond this rudimentary knowledge, tRNA is generally not on the mind of most research scientists, unless they meet it through chance encounter.

Recently, however, tRNA is becoming a prominent research subject once again due to many new discoveries. The consequences of tRNA overexpression on gene expression in human diseases such as cancer have now been well documented.^1–4^ Human mitochondrial tRNA mutations are a huge source of a wide range of human diseases,^5^ including MELAS (mitochondrial encephalomyopathy, lactic acidosis, and stroke-like episodes)^6^ and MERRF (myoclonic epilepsy with ragged-red fibers).^7^ Mutations in many tRNA modification enzymes have been linked to human diseases^8–12^ and many tRNA modifications are receiving renewed interest as increasing numbers of human tRNA modification enzymes become identified, thus enabling focused studies of specific tRNA modifications. Recent discoveries implicate tRNA fragments as small RNAs that affect many cellular processes,^13–16^ are involved in human diseases^17–19^ and even directly in epigenetic inheritance in germ-line cells.^20,21^ Thus tRNA molecules provide a reservoir of RNA species whose function extends beyond translation.

This review focuses on genome-wide aspects of the function of human tRNAs and consequences of their modifications on decoding. I refer to several recent, excellent reviews on the roles of tRNAs in other organisms, the functions of mitochondrial tRNAs, modification enzymes and diseases, and tRNA fragments.^22–28^

## Human tRNA gene features

When the human genome was sequenced in 2001–2003, tRNA genes could be readily identified computationally because a canonical tRNA gene contains highly conserved residues located at predictable distances from each other. This revealed the number of tRNA genes in the reference human genome to be 610 according to the genomic tRNA database (hg19 version)^29^ although additional tRNA genes could be found in the human population.^30^ This gene copy number is not unusual among eukaryotic genomes, which generally contain from a few hundred to a few thousand tRNA genes. However, two completely unexpected features of human tRNA genes were found: a large sequence diversity of the tRNA genes and the clustering of tRNA genes at specific chromosomal locations.

**Fig. 1:**
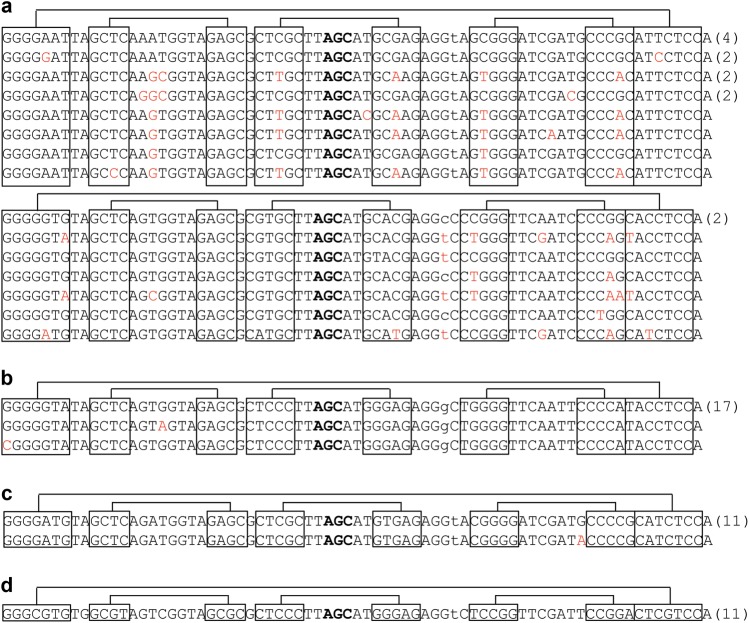
tRNA^Ala^(AGC) gene sequence diversity. Only genes with tRNAScan score >50 are shown. The four helices are connected by lines. Anticodon nucleotides are marked in bold. The number of genes with identical sequences is in parenthesis. The sequence difference compared to the tRNA with the highest number of gene copies is in red. **a** The human reference genome has 22 genes, 15 sequences. **b** *C. elegans* has 19 genes, 3 sequences. **c** *D. melanogaster* has 12 genes, 2 sequences. **d** *S. cerevisiae* has 11 genes and 1 sequence

Whether a gene truly encodes a tRNA may reflect the secondary structure of its gene product. tRNA folds into a cloverleaf secondary structure, which requires a sufficient number of Watson–Crick or G-U wobble pairs in the four helical stems. The propensity of a tRNA sequence to fold into this secondary structure is reflected in the tRNAScan score,^31^ assigned for each tRNA gene sequence in the genomic tRNA database. As a rule of thumb, a tRNA gene with a score of >50 can be considered to fold into this secondary structure. tRNA genes with a score of <50 may have too many base pair mismatches in their helical stems and therefore can  fold into alternative secondary structures. Indeed, it was shown that a low-scoring tRNA^Asp^ (score 28.6) folds into a non-tRNA structure and cannot be aminoacylated. However, this “tRNA” interacts with a specific mRNA in cells to affect the alternative polyadenylation of this mRNA.^32^ When only human tRNA genes with a score of >50 are taken into account, 264 different sequences were identified among 423 potential human tRNA genes,^29^ representing a gene/sequence ratio of 1.6. In comparison, the tRNA gene count and sequences in the *Saccharomyces cerevisiae* genome are 272 (score >50) and 53, respectively, representing a gene/sequence ratio of 5.1. Therefore, human tRNA genes are much more diverse in sequence than either their yeast counterparts or the tRNA genes of two other model organisms, *Caenorhabditis elegans* and *Drosophila melanogaster* (Fig. 1). This high tRNA gene sequence diversity is conserved in all mammalian genomes, suggesting that the diversity was already present upon the evolutionary emergence of the mammals.

tRNA genes with the same anticodon and different body sequences are termed “isodecoders”.^33^ The more commonly known term of tRNA “isoacceptors” refers to tRNA genes with different anticodons and charged with the same amino acid. In the example shown in Fig. 1a, all 15 human tRNA isodecoders belong to a single-isoacceptor family of Ala (AGC). It is clear that not all isodecoder genes are expressed in every human cell, and isodecoder expression is expected to vary widely in human tissues.^34,35^ Direct sequencing of all tRNAs by the DM-tRNA-seq approach (see below) identified ~200 different tRNA sequences (some with tRNAScan score <50) that are present within a 1000-fold molar range in HEK293T cells.^36^

tRNA isodecoder sequences are highly conserved in the mammalian genomes, suggesting that they serve important functions. However, a large number of tRNA isodecoders may be more harmful than useful. Each tRNA isodecoder in cells is aminoacylated (charged) by its cognate aminoacyl-tRNA synthetase with the correct amino acid according to a set of “identity” elements in the tRNA. At the same time, each tRNA isodecoder needs to avoid being mischarged by the 19 other tRNA synthetases as this could result in potentially harmful levels of mistranslation; it therefore should also carry “preventive” elements against mischarging. Changes in a tRNA sequence that produce a new isodecoder may therefore reduce or eliminate a preventive element for mischarging.

Using a green fluorescent protein reporter that contains an UAG stop codon, different tRNA isodecoders have been analyzed for their comparative stop codon suppression efficiency in HeLa cells.^37^ Intriguingly, the stop codon suppression efficiency of human tRNA^Ser^ isodecoders varied over a 10-fold range. Furthermore, a small change in a tRNA^Ser^ isodecoder sequence increased the suppression efficiency by another 3-fold, suggesting that the natural isodecoder sequences are not conserved for maximizing their utility in translation, at least in the context of stop codon suppression.

**Fig. 2:**
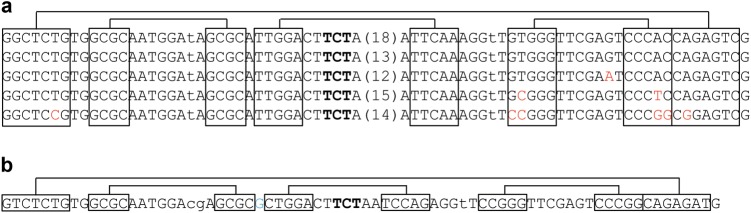
Human tRNA^Arg^(TCT) gene sequences. The four helices are connected by lines. Anticodon nucleotides are marked in bold. The number of genes with identical sequences is in parenthesis. **a** Five “house-keeping” isodecoder genes. All have introns shown in parenthesis with the number of intronic nucleotides indicated. Sequence differences are in red. **b** The central nervous system isodecoder gene. It has no introns. Also shown in blue is the nucleotide between the D stem and anticodon stem, and it is a G in this isodecoder but A in all others

The best-characterized tRNA isodecoder so far was from the tRNA^Arg^(UCU) family that reads the AGA codon of arginine.^38^ This family has five genes in mice and six genes in humans. Both human and mouse have one isodecoder (Tr-20 in mouse) that is very different from the other isodecoders in both sequences and the presence of introns (Fig. 2). The mouse Tr-20 isodecoder is primarily expressed in the central nervous system (CNS) where it accounts for ~60% of all tRNA^Arg^(UCU) expression, whereas the other isodecoders are expressed in all tissues. A genomic mutation in mice that significantly reduced the expression of the Tr-20 isodecoder increased ribosome pausing specifically at AGA codons and resulted in neuro-degeneration, likely due to increased protein misfolding or degradation. These findings point to the differential, tissue-specific functions of isodecoder tRNAs.

The second unexpected feature of human tRNA genes is the clustering of 158 genes (144 with a tRNAScan score >50) within just 2.67 million base pairs immediately next to the class I major histocompatibility complex (MHC, aka HLA) genes on chromosome 6.^39^ Class I MHC proteins present peptide antigens in nucleated cells for surveillance by immune cells and the class I MHC gene sequences are the most polymorphic in the human population.^40^ The chromosomal association of approximately one third of all human tRNA genes with the MHC therefore may suggest the somewhat unlikely possibility that tRNAs contribute to antigen presentation and are part of the human immune system. The chromosome 6 region where these tRNA genes reside is also the home for the genes that encode many histone proteins and zinc-finger transcription factors. An alternative model for the function of this tRNA cluster is to keep the chromatin structure in this region distinct through high-level RNA polymerase III transcription, to facilitate the high-level transcription of these protein-coding genes. To my knowledge, there is currently no evidence for direct, functional association of tRNA with antigen presentation, and the hypothesis of linking tRNA gene transcription to facilitate RNA polymerase II transcription in this region remains to be tested.

## Human tRNA modifications

**Fig. 3:**
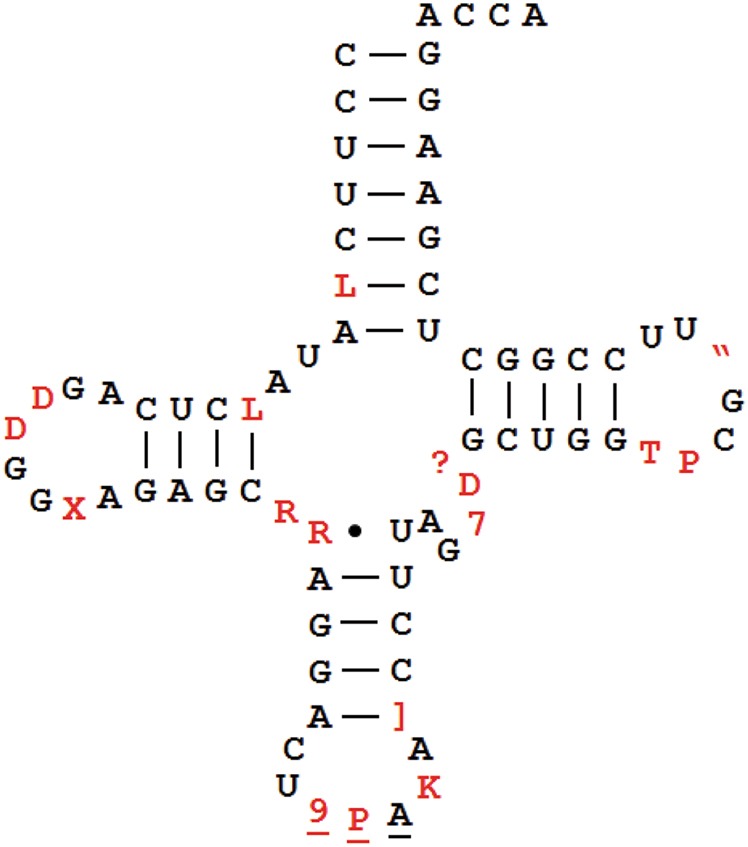
Human cytosolic tRNA^Tyr^ has the most known modifications. Modified nucleotides are in red; anticodon nucleotides are underlined. Single letter and symbol designations are the nomenclatures used by the RNA modification databases such as Modomics.^108–110^ L *N*^2^-methyl-G (m^2^G), D dihydro-U, X 3-(3-amino-3-carboxypropyl)-U (acp^3^U), R *N*^2^,*N*^2^-dimethyl-G (m^2^_2_G), 9 galactosyl-queuosine (gal-Q), P pseudoU (Ψ), K *N*^1^-methyl-G (m^1^G);], *N*^1^-methylpseudo-U (m^1^Ψ), 7 *N*^7^-methyl-G (m^7^G), ? 5-methyl-C (m^5^C), T 5-methyl-U (m^5^U), “ *N*^1^-methyl-A (m^1^A)

Like all mammals, humans have chromosomal or nuclear-encoded tRNA genes that produce tRNAs for cytosolic translation and mitochondrial-encoded tRNAs genes that produce tRNAs for mitochondrial translation. Some mitochondrial tRNA sequences are also present in the human chromosome, and these nuclear-derived tRNA transcripts may be imported into mitochondria.^29,41^ Not all human tRNA genes are transcribed in all cells,^34,35^ and tRNA expression is tissue specific.^2,42^ The nuclear-encoded tRNAs are very extensively modified, containing an average of 13 modifications per molecule. Individual tRNAs are, however, modified unevenly; for example, tRNA^Tyr^ from placenta has 17 modifications^43^ (Fig. 3), whereas tRNA^Sec^ from HeLa cells has only 3.^44^ Mitochondrial tRNAs are generally modified to a lesser extent, containing on average of five modifications per molecule. Since one function of tRNA modification is to increase the stability and rigidity of the tRNA, the presence of fewer modifications in mitochondrial tRNAs contributes to their lower stability and their reliance on specific modifications for structural integrity.^45^

Comprehensive mapping of tRNA modifications is laborious, as one first needs to purify a specific tRNA at appreciable amounts. The current method for tRNA purification uses affinity chromatography with a complementary DNA oligonucleotide linked to a solid resin or bead. Purified tRNAs are digested with ribonucleases (e.g., T1 that cuts 3′ of guanosine) to generate oligonucleotides whose modifications can be precisely identified by mass spectrometry.^46–49^ This approach works very well and has, for example, led to the complete determination of the modifications of all 22 mitochondrial tRNAs in a mammalian cell.^50^ Owing to the large sequence diversity of the nuclear-encoded human tRNA isodecoders, the full mapping of cytosolic tRNA modifications has not yet been accomplished because the oligonucleotide affinity columns generally do not have sufficient resolution to allow purification of all individual isodecoders. However, the total modification content of the total cellular or compartmental tRNAs can be quantified upon isolating total tRNA from whole cells or specific cellular compartments followed by nuclease digestion to mononucleosides and mass spectrometry. Importantly, such analyses of modification content have identified condition-dependent tRNA modifications in response to stresses.^51,52^ In this example, the functional significance of such differential modification was demonstrated by the finding that tRNA^Leu^(CAA)-specific 5-methyl-cytosine (m^5^C) modification in the wobble anticodon position enhanced translation of stress response genes.^53^

Analysis of total tRNA modifications indicates that many tRNA modifications are incomplete under physiological conditions. This variation in the levels of tRNA modification should enable cellular adaptation to environmental changes. Therefore, dynamic tRNA modifications seem likely to play similar types of roles in cellular regulation in an analogous manner to dynamic mRNA modifications such as *N*^*6*^-methyladenosine (m^6^A).^54^ It is therefore of high biological interest to measure the extents of tRNA modification at individual sites. For human mitochondrial tRNAs that can be purified individually, the fractional extent of modification can be determined by high-resolution and quantitative mass spectrometric analysis of oligonucleotides generated by nucleases.^53,55^

**Fig. 4:**
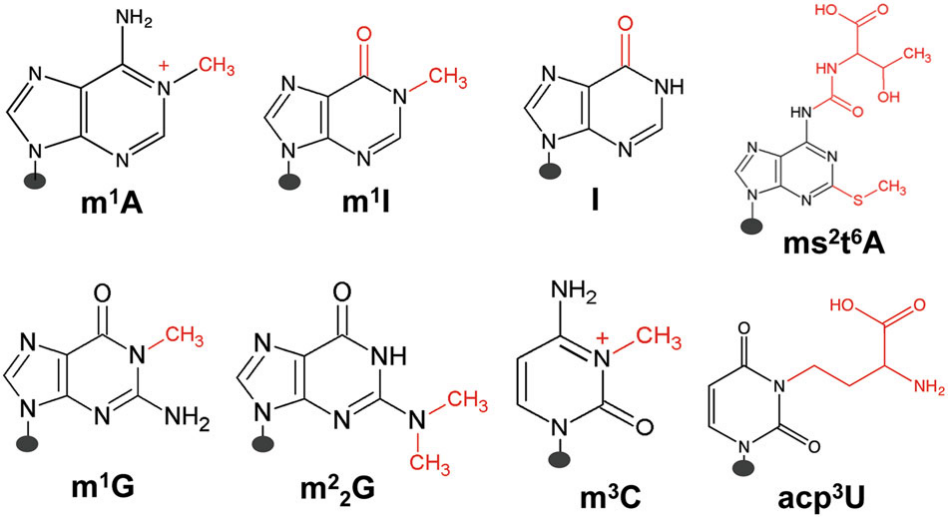
Human nuclear-encoded tRNA modifications investigated by mutation and stop signatures in demethylase-tRNA-seq (DM-tRNA-seq). m^1^A *N*^1^-methyl-A, m^1^I *N*^1^-methyl-inosine, I inosine, ms^2^t^6^A 2-methylthio- *N*^*6*^-threonylcarbamoyl-A, m^1^G *N*^1^-methyl-G, m^2^_2_G *N*^2^,*N*^2^-dimethyl-G, m^3^C 3-methyl-C, acp^3^U 3-(3-amino-3-carboxypropyl)-U

Certain types of tRNA modifications and their extent can be semiquantitatively determined by high-throughput tRNA sequencing. Methylations at the Watson–Crick face (Fig. 4) do not always lead to full reverse transcriptase (RT) stops in cDNA synthesis; rather, different RTs can read through these modifications at varying efficiencies, leaving behind mutation signatures.^56–58^ The mutation signatures can be highly dependent upon the modification and the sequence context, as with *N*^*1*^-methyladenosine (m^1^A).^59^ The mutation signatures in cDNA synthesis from known tRNA modifications have been used for machine-learning algorithms to identify new modifications in tRNAs and even in mRNAs.^57,60^

Using a highly processive, thermophilic RT (TGIRT)^61^ for tRNA sequencing, we found that the mutation and stop fractions at several Watson–Crick face methylations can be used to semiquantitatively determine the modification fraction at specific sites^58,62^ (Fig. 4). The commonly used Superscript RT in Illumina sequencing stopped at high levels at modifications such as the m^1^A present in almost all human cytosolic tRNAs. The high level of stops made it more difficult to obtain sequencing reads that pass the m^1^A modification. TGIRT read through these modifications much more efficiently leaving behind mutations at the modified nucleotide; the efficient read-through also enabled the assessment of multiple modifications in the same tRNA molecule. Our sequencing library avoided 5′ adapter ligation before cDNA synthesis, so that the fraction of RT that stops at each tRNA position could also be measured. We used the term “modification index” (MI) to describe the sum of the mutation and stop fraction at each site.^58^ We found that the MI value approximated the modification fraction very well as validated by a gel-based method that measured stops in cDNA synthesis using an RT with high-stop fractions (AMV RT). In addition, we were able to quantify the modification from more elaborate Watson–Crick face modifications such as 2-methylthio-*N*^*6*^-threonylcarbamoyl-A (ms^2^t^6^A)^63^ by measuring the stop fraction alone.

The main advantage of sequencing-based methods is that the assessable modifications can be determined simultaneously at single-base resolution potentially for most, if not all, isodecoders. The main disadvantage is that only some modification types can be analyzed in this way. We estimate that our tRNA-seq-based method currently can assess about a quarter of all human tRNA modification sites.^58^ Modifications that cannot yet be assessed currently include some of the most abundant tRNA modifications such as pseudouridine (Ψ) and 5-methylcytosine (m^5^C) or some bulky modifications such as *N*^*6*^-threonylcarbamoyladenosine (t^6^A) and 5-methoxycarbonylmethyl-2-thiouridine (mcm^5^s^2^U).

**Fig. 5:**
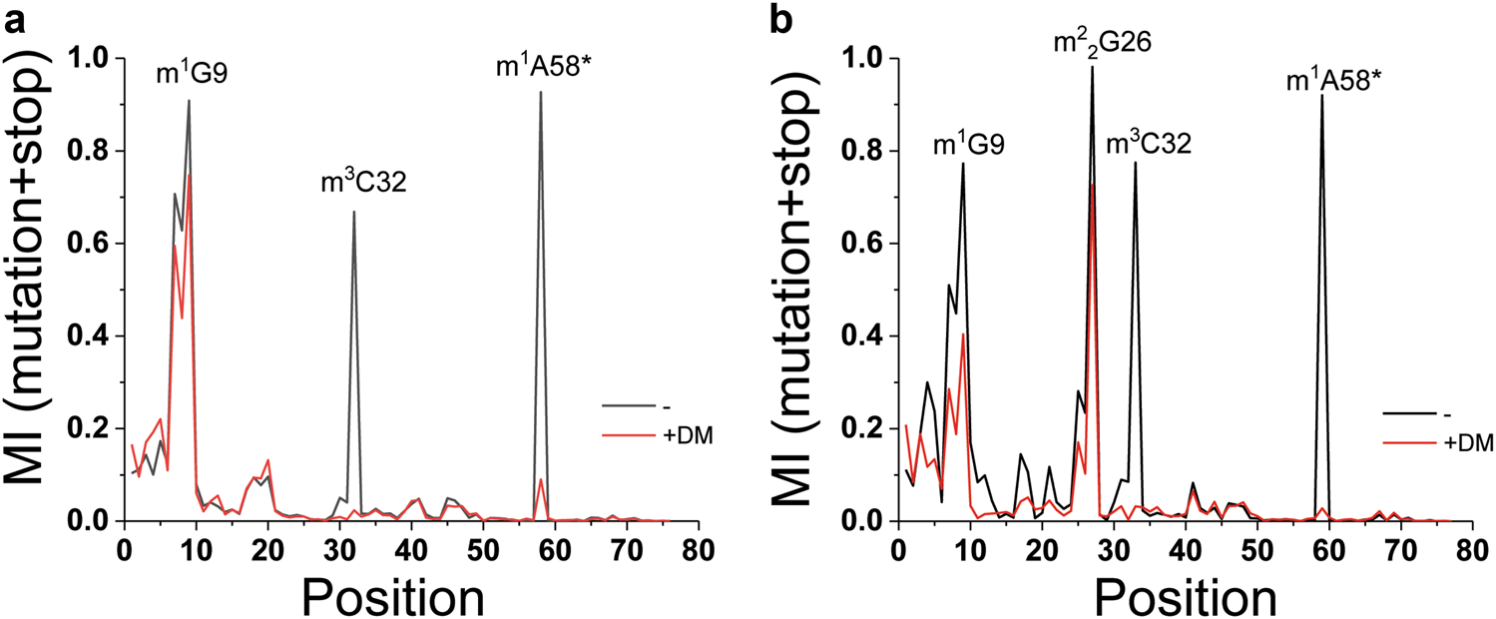
Watson–Crick face methylations in tRNA^Arg^(UCU) isodecoders. Data from ref. ^58^. +DM demethylase-treated, m^1^A58* only mutation signature was measured for this modification due to the challenge of mapping stops derived from short reads. **a** An isodecoder from the “house-keeping” group of five intron-containing genes. **b** The single, central nervous system-specific isodecoder

An example of applying TGIRT-based tRNA sequencing to discover new modification in a specific isodecoder is shown in Fig. 5. As discussed above, the tRNA^Arg^(UCU) genes are expressed in a “house-keeping” group of 4–5 genes present in all cells and in a “CNS” group of a single gene only expressed in the CNS in the mouse. The “CNS” tRNA^Arg^(UCU) isodecoder can also be found in minute amounts in the HEK293T cells by tRNA sequencing.^62^ To identify new modifications in an isodecoder, the MI value for each tRNA residue from sequencing reactions with or without demethylase treatment was calculated. In this case, without demethylase treatment, three high MI peaks were readily seen for a “house-keeping” isodecoder (Fig. 5a). The MI values for all three peaks decreased upon demethylase treatment, indicating that these corresponded to Watson–Crick face methylations that could be removed by the demethylases; in this case, they corresponded to the predicted m^1^A58, m^3^C32, and m^1^G9 modifications. For the “CNS” isodecoder, one additional high peak was present, and the MI for this peak also decreased upon demethylase treatment (Fig. 5b). This new peak is interpreted to correspond to an *N*^*2*^, *N*^*2*^-dimethyl-G (m^2^_2_G) modification present in many other human tRNAs at this same location. This approach shows that the “CNS” tRNA isodecoder not only has distinct sequences compared to the “housekeeping” isodecoders but also contains an additional modification, which may contribute to its unique role in neuronal translation.

The function of tRNA modifications is generally direct in mRNA decoding in the case of modifications located in the anticodon loop, but other properties can also be affected including stability, localization, ribosome binding, and translational dynamics. For the decoding role, the strongest effects are derived from modifications to the wobble anticodon (position 34 in the tRNA nomenclature) and to the residue to the immediate 3′ of the anticodon nucleotides (position 37). A-to-Inosine (I) modification occurs in at least eight human tRNA wobble positions that play an obvious role to expand the base pairing capability from A34-U to I34-U/I34-C and maybe even I34-A.^64^ 2′O-methylation of G34/C34 (Gm/Cm) is another common wobble modification in human tRNAs, and it is likely to enhance codon–anticodon interactions on the ribosome.^10,65^ Elaborate U34 modifications, such as 5-methylaminomethyl-2-thiouridine (mcm^5^s^2^U), can confer decoding bias for the cognate codons.^66,67^ Not all known nucleotide 34 modifications have well-defined functions. For example, the human ALKBH8 is a member of the ALKB homolog family that includes the mRNA m^6^A demethylases ALKBH5 and FTO. It has a methyltransferase domain that methylates 5-carboxymethyl-U (cm^5^U) to 5-methoxycarbonylmethyl-U (mcm^5^U)^68,69^ and a hydroxylation domain that hydroxylates mcm^5^U34 in tRNA^Gly^(UCC) to 5-(carboxyhydroxymethyl) methyl ester-U (mchm^5^U).^70,71^ The methylation reaction can also be carried out by another human enzyme, hTRMT9, and plays a role in enhanced decoding,^72^ whereas the function of the hydroxylation is not known. Nucleotide 37 is an A or G in tRNA, and its modifications are always at the Watson–Crick face. G37 modifications are typically N1-methylguanosine (m^1^G) and can be further modified to peroxywybutosine (W) in tRNA^Phe^.^73^ A37 modifications can be *N*^*6*^-threonylcarbamoyladenosine (t^6^A) or *N*^*6*^-isopentenyladenosine (i^6^A) with large chemical groups at the *N*^*6*^-position. A major function of the A/G37 modification is to prevent frameshifting.^74–77^

**Fig. 6:**
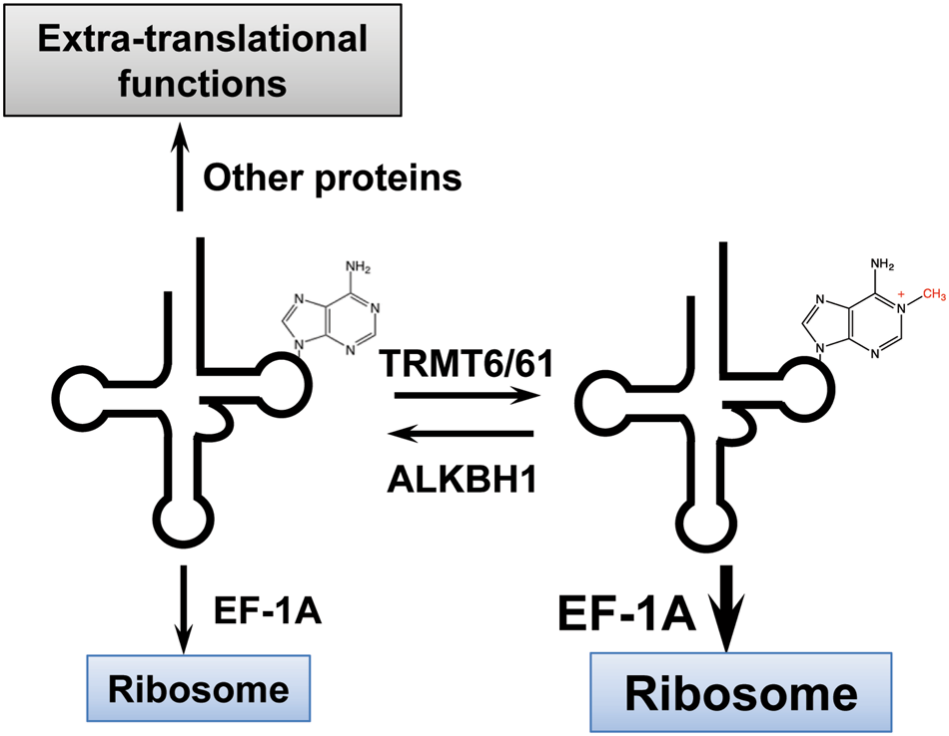
Working model for tRNA m^1^A58 demethylase (eraser) function. Modified tRNA has a higher affinity for the EF-1A protein, which delivers tRNA to the ribosome. Elevated ALKBH1 activity decreases tRNA availability for translation but may enhance tRNA binding to other cellular proteins due to reduced competition by EF-1A

Modifications outside of the anticodon loop can perform many other tRNA-related functions. For base methylations alone, m^1^A and m^1^G9 between the acceptor and D-stem help fold mitochondrial tRNAs into their correct structures.^78,79^ This modification in the cytosolic tRNAs can also increase tRNA rigidity through enhanced base stacking.^80,81^ Similarly, the m^2^_2_G26 modification between the D and anticodon stems increases the stiffness of the modified tRNA, which is likely to be important when tRNA on the ribosome needs to accommodate the conformational dynamics of the ribosome during translational elongation.^82,83^ Perhaps the best-studied modification outside of the anticodon loop is m^1^A58 located in the T loop of tRNA. M^1^A58 adds a positive charge to the nucleobase. It is present in almost all human tRNAs^58,84^ and is essential for the stability of the initiator tRNA^Met^.^85^ Interestingly, the m^1^A58 modification fraction on individual tRNAs can be variable in different cell types and under different cellular conditions.^55,86^ The fractional modification is likely maintained by the combined actions of the m^1^A58 methyltransferase, TRMT6/TRMT61A^87^ or TRMT61B,^88^ and the m^1^A58 demethylase, ALKBH1 in the cytosol^86^ and in mitochondria.^55^ A working hypothesis (Fig. 6) for this reversible modification for the cytosolic tRNA is that the m^1^A58-modified and unmodified tRNAs have differential affinity for the elongation factor EF1A. EF1A delivers tRNA into the A-site of the ribosome in protein synthesis. By adjusting the level of this single modification in specific tRNAs, cells can readily titrate the amount of these tRNAs available for translation through altering their affinity for EF1A and thus for the ribosome. Hypo-modified tRNAs that escape EF1A binding may bind other cellular proteins to coordinate translation and other cellular processes (see below). Although ALKBH1 is the only known “eraser” for tRNA so far, other human enzymes will likely be found that act on other types of tRNA modifications.

## Functional genomics

Translational regulation occurs at the initiation, elongation, and termination steps. Elongation is the step at which tRNA decoding can play the most fundamental roles. In total, 18 of the 20 amino acids have more than one codon in the genetic code and the distribution of codon usage varies widely in the coding sequences among different organisms. Because multiple codons are present for many amino acids, multiple tRNAs with distinct anticodons (i.e., isoacceptors) are needed to read these codons. In humans, tRNA isoacceptors are present for 12 amino acids, ranging from two (Glu, Lys, Gln), three (Ile, Val, Thr, Ala, Gly, Pro), four (Ser) to five (Leu, Arg). For the six amino acids (Phe, Tyr, His, Asn, Asp, Cys) that have just two codons each, a single tRNA is present in the genome. Here the wobble anticodon nucleotide of all these tRNAs, with the exception of tRNA^Cys^, can be modified to more efficiently decode both codons. One additional tRNA property used in decoding is aminoacylation or charging. A charged tRNA has a covalently attached 3′ amino acid and is required for protein synthesis.

**Fig. 7:**
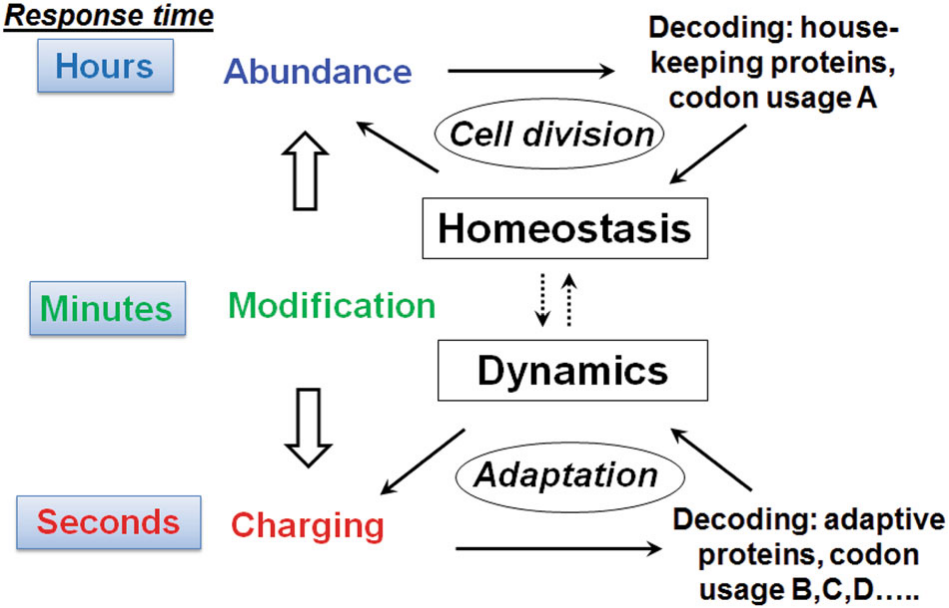
Dynamics of protein synthesis needs and tRNA response. During cell cycle progression, most translation activity is for the synthesis of highly abundant house-keeping proteins (~10–20% of all genes), and the tRNA abundance matters most. In stressful times or environmental fluctuations, translation activity switches to the synthesis of response or regulatory proteins (30–50% of all genes), to which tRNA charging significantly contributes. Modification is the “wild card” that tunes the regulation under all conditions

The three properties of tRNA—abundance, modification, and charging—are coordinated in protein synthesis according to cell states and conditions (Fig. 7). Translation is most active during the cell cycle when the cell doubles its protein mass. Under these conditions, most protein synthesis activity is devoted to the synthesis of the highly abundant house-keeping proteins, such as the ribosomal and cytoskeletal proteins. Here the abundance of tRNAs, and particularly the representation of tRNA isoacceptors, plays the most important role in translational regulation by accommodating the synthesis of those house-keeping proteins representing ~10–20% of human genes. Under these conditions, most tRNAs are highly charged,^36^ and the modification levels for most tRNAs are high, at least in the case of the Watson–Crick face methylations.^58^

**Fig. 8:**
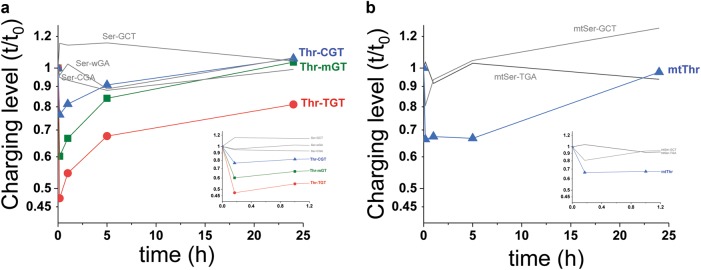
tRNA-charging level changes upon threonine starvation. Starvation of MDA-MB-231 cells was performed by exchanging the growth medium with the same medium lacking the amino acid threonine at *t* = 0. Total RNA was isolated at the indicated time points under mild acidic conditions (pH ~ 5) where tRNAs remained aminoacylated. Relative charging levels were measured using tRNA microarrays at the isoacceptor resolution.^89, 111^ **a** Nuclear-encoded tRNA^Thr^ and tRNA^Ser^. Thr-mGT corresponds to an array probe complementary to all Thr-AGT and three of the five Thr-CGT *tRNA* genes. Thr-CGT corresponds to two of the five Thr-CGT *tRNA* genes. **b** Mitochondrial-encoded tRNA^Thr^ and tRNA^Ser^. Inset shows the expanded view for early time points. Upon starvation, charging levels for all tRNA^Thr^ isoacceptors immediately dropped but to different degrees. Differential charging remained for several hours. Mitochondrial tRNA^Thr^ recovered more slowly than the cytosolic tRNA^Thr^. Charging levels did not drop for tRNA^Ser^

When cells are stressed or exposed to conditions where cell cycle progression is halted or severely slowed, the major protein synthetic activity switches from the production of house-keeping proteins to stress response or other regulatory proteins. Under stress, the level of charged tRNAs can rapidly change. Importantly, the charging levels among the tRNA isoacceptors for the same amino acid can become different and so become a useful parameter for the stress response or production of adaptive proteins for the new condition. At the transcriptomic level, selective or differential tRNA isoacceptor charging was first demonstrated directly in *E. coli* under amino acid starvation,^89^ which has significant consequences on the translational efficiency of mRNAs for many native *E. coli* proteins.^90^ Selective tRNA isoacceptor charging is derived in part by cells overexpressing certain isoacceptors beyond their needs for translating house-keeping proteins.^91^ These overproduced tRNA isoacceptors can remain at higher charging levels when stress or other changes to the environment halt the synthesis of house-keeping proteins. In a human cell line, starvation of an essential amino acid also leads to differential charging among tRNA isoacceptors for the corresponding starved amino acid (Fig. 8).

Human tRNA modifications can also change reflecting culture conditions with different concentrations of glucose.^86^ Glucose deprivation increased the expression of the m^1^A58 eraser protein ALKBH1, resulting in decreased m^1^A levels for several tRNAs. As the m^1^A58 modification increased the effective concentration of tRNA for translation, a reduction of m^1^A58 levels decreased translation under low-glucose conditions. This study demonstrates that tRNA modification levels can respond to distinct culture conditions to adapt to the efficacy of translation.

The three main properties of tRNA exhibit dynamic changes with a distinct time scale (Fig. 7). Mature mammalian tRNAs are extremely stable with a half-life on the order of 100 h.^92^ Based on cellular tRNA/ribosome ratios (~10 on average) and protein synthesis rate on the ribosome (~2–5 peptide bonds per second on average), charged tRNAs should turn over every few seconds in a human cell when protein synthesis is fully active as occurs, for example, during cell cycle progression. Although tRNA modification dynamics remain to be determined in human cells, the presence of eraser enzymes would enable demodification reactions within a few minutes or less, as is typical for enzymatic reactions on macromolecules. The distinct dynamic range of distinct tRNA properties from seconds to hours or even days enables tRNAs to behave as either rapidly or slowly reacting responders to protein synthesis under wide ranging conditions.

**Fig. 9:**
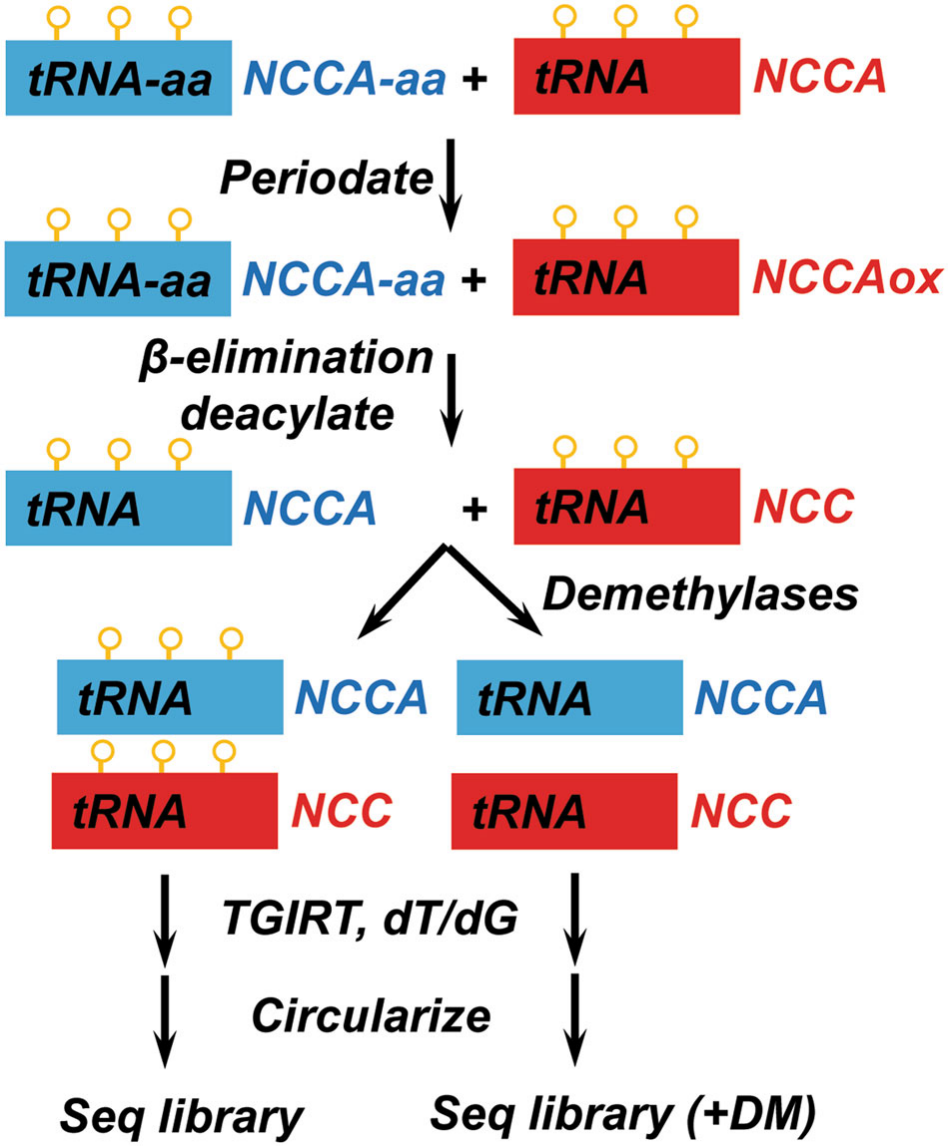
DM-tRNA-seq can measure tRNA abundance, modification, and charging in a single-sequencing library. Periodate only oxidizes uncharged tRNA. β-Elimination removes the oxidized 3′ nucleotides and deacylates charged tRNAs at the same time. The sample is then split into two, and one part treated with demethylases to remove m^1^A, m^3^C, and m^1^G (orange circles). Both samples are subjected to cDNA synthesis by a processive RT with both dT/dG-ending template-switching primers in case of TGIRT. cDNAs are circularized, followed by PCR to build sequencing libraries

Our efforts to develop high-throughput sequencing methods for tRNA now allow the measurement of all three tRNA properties in a single-sequencing reaction^36,58,62^ (Fig. 9). As mentioned above, the DM-tRNA-seq approach combines enzymatic demethylation and a processive thermophilic RT for measurement of tRNA abundance and modification. Extending this method to tRNA charging involves a periodate oxidation/β-elimination reaction of the total RNA before demethylase treatment. This chemical reaction removes the 3′A residue in all uncharged tRNAs, while charged tRNAs remain intact. Since all tRNAs end with 3′CCA, uncharged tRNAs end with 3′C, while charged tRNAs end with 3′A in this “one-pot” sequencing reaction. This method is applicable to any RNA sample from cell lines and tissues.

## Extra–translational tRNA–protein interactions

At a cellular concentration of tens of millions of copies (up to 100 µM) per cell, tRNAs are well suited for interactions with proteins not directly involved in protein synthesis, even at µM affinity. The GCN2 kinase–tRNA interaction is perhaps the best-studied system that serves an extratranslational function for tRNA.^93–97^ GCN2 is a protein kinase with a kinase domain that phosphorylates eIF2α and a regulatory domain that senses uncharged tRNA levels to activate the kinase domain. eIF2α is a conserved eukaryotic initiation factor involved in delivering the initiator tRNA^Met^ to the scanning 40S ribosome. Phosphorylated eIF2α is inactive; therefore, the phospho-eIF2α level regulates the global translation activity. Proliferating cells have full translation activity and little uncharged tRNAs are available to activate the GCN2 kinase. Under stress or nutrient limitation, the uncharged tRNA level increases, which results in GCN2 activation and increased eIF2α phosphorylation either through a direct tRNA–GCN2 interaction or mediated through the ribosome. This decreases the global translation activity in time of stress or nutrient limitation.

Cell apoptosis can be triggered by the mitochondrial release of the cytochrome C protein to the cytosol that activates caspases. tRNA can bind the cytochrome C protein to block its binding to the caspase activator Apaf-1.^98^ tRNA binding prevents caspase activation under conditions where spurious disruption of mitochondria would not result in apoptosis, thereby ensuring that apoptosis only takes place when the cytosolic cytochrome C concentration has reached a certain threshold.

The interferon response to infection is mediated by the interferon-induced tetratricopeptide repeat (IFIT) protein family. The IFIT5 protein binds tRNA and may modulate the activity of the RIG-I protein, a cellular sensor for viruses.^99,100^ The cellular HIV restriction factors Sfn11 and Hili bind tRNA to reduce HIV virus replication.^101,102^ Both proteins reduce the translation of the HIV Gag protein in a codon usage-dependent manner. Since high levels of Gag protein translation are required for HIV replication, and because HIV genes have codon usages distinct from human genes, the interaction of these proteins with tRNA may selectively reduce the available amount of “rare” tRNAs to below a threshold for efficient Gag protein translation.

My laboratory has developed a systematic approach to identify protein candidates that interact with tRNA in cells.^103^ Our approach began with a computational, machine-learning analysis in which the known crystal structures of tRNA–protein complexes were used to compose Chemical Context Profiles (CCP). The CCP value scores the relevance of individual amino acid and nucleotide functional groups in the RNA–protein interaction interface. To identify candidates of new tRNA-binding proteins, 100,000 three-dimensional (3D) tRNA–protein decoys were built for each of >800 mammalian proteins or protein domains in the protein database. These 3D models were then scored according to the CCPs obtained from the learning set above. This approach identified 37 mammalian proteins as candidates for tRNA binding; none was previously known to be an RNA-binding protein. Using the ultraviolet crosslinking and immunoprecipitation approach^104^ commonly used in the field to study protein–RNA interactions, we experimentally validated tRNA binding for six of these predicted proteins in HEK293T cells.

One of the new tRNA-binding proteins is the mitogen-activated protein kinase kinase (MEK), which is required for cell cycle progression. We further investigated the property and potential function of the MEK–tRNA interaction in vitro and in several cell lines.^105^ We found that tRNA inhibits MEK2 kinase activity, consistent with our working hypothesis that tRNA provides an interface between translation activity and cell cycle progression. Cell cycle progression requires high levels of translation because of the synthesis of the very abundant house-keeping proteins. High-level translation utilizes most of the cellular tRNAs so that only a small amount of non-ribosomal-associated tRNAs would be available for interaction with other proteins. When translation activity decreases for any reason, more “free” tRNA becomes available for interacting with proteins that regulate other cellular processes. The tRNA-mediated inhibition of MEK protein kinase activity would slow down cell cycle progression, enabling cells to wait until translation can resume at full speed.

## Future perspectives and challenges

A major challenge in human tRNA biology is to elucidate the function of tRNA isodecoders. The conservation of isodecoder genes in the mammalian genomes and the demonstration of a CNS-specific function of the tRNA^Arg^(UCU) isodecoder indicate that many isodecoders are likely expressed uniquely in different tissues and cell types. Specific tRNA isodecoders could enhance cell type-specific translation in a codon or even mRNA-dependent manner. Other tRNA isodecoders may show preferred interactions with other cellular proteins for extratranslational functions.

Another major future challenge is the complete mapping of human tRNA modifications and assignment of their roles in biological regulation. As of today, <50% of nuclear-encoded human tRNAs have their modifications mapped in just one cell type. The complete modification map of the other human tRNAs remains to be charted. For each tRNA, the modification fraction at some sites may vary in a cell type- and cell state-dependent manner. How dynamic changes of tRNA modifications affect different biological processes remains to be determined.

A third challenge is to reveal how cells integrate the three tRNA properties of abundance, modification, and charging to achieve robust regulation of translation under all conditions. Translational regulation involves the temporal and spatial interplay between all three tRNA properties. So far, the primary focus has been on the role of tRNA abundance in translation. How rapidly changing charging levels and the dynamics of tRNA modification complement tRNA abundance to achieve optimal regulation remains to be determined.

Although not covered in this article, tRNA fragments have become increasingly important in a wide range of biological processes. These small RNAs are derived from the conditional cleavage of mature tRNAs or tRNA precursors by cellular nucleases. Given the extremely high levels of tRNA in a human cell, converting just a few percentage of mature tRNA into fragments would generate millions of small tRNA-derived molecules that can directly interact with cellular proteins or mRNAs. How much a role modifications play in tRNA fragment biology is a fascinating question that is only beginning to emerge.

Finally, the field requires a major technological advance to carry out single-cell tRNA sequencing. Single-cell transcriptome sequencing has become a mainstream for biological studies and has uncovered many surprising cellular physiologies such as new subtypes of cells in organs that are previously considered homogenous.^106,107^ Currently, tRNA-seq is much more difficult to carry out than mRNA-seq and so facilitating such studies would be a major technical achievement. The effort should well be worth it, as single-cell tRNA-seq may reveal unique expression and/or modification patterns that reflect regulatory mechanisms to adjust demand for protein synthesis in individual cells.
